# Supplementary material for: Comparison of respiratory symptoms and pulmonary functions of adult male cigarette smokers and non-smokers in Sri Lanka; A comparative analytical study
Source: PLoS One. 2025 Feb 12;20(2):e0315095. doi: 10.1371/journal.pone.0315095 (PMC11819500; doi:10.1371/journal.pone.0315095)
Supplement: Attachment — Submitted filename: Plos 1 Comparison of Respiratory Symptoms and Pulmonary Functions of Adult Male.docx [file pone.0315095.s001.docx]

Title: Comparison of Respiratory Symptoms and Pulmonary Functions of Adult Male Cigarette Smokers and Non-Smokers in Sri Lanka; A Comparative Analytical Study

The manuscript addresses a very important public health issue globally. This study is carried out in Th Sri Lanka and compares respiratory health of non-smoking and smoking individuals. Study design is a descriptive cross-sectional study and attempts to fill the existing gap in the research evidence as regards to effect of smoking on pulmonary function in Sri Lanka

The methodology applied int eh study is appropriate for the aim of the study. and the research question is clear.

The manuscript would benefit from increased clarity in the methods description and the style of writing overall. Currently the study is described with fairly minimal detail. I do think that adding the actual details, particular related to the methods applied to carry out the study, would make the manuscript stronger. Also I recommend arranging the discussion and specific paragraphs in the more logical way to help to keep message of the manuscript clear.

Specific edits.

I have highlighted in yellow the particular problem within the text clipping and added the comment below to aid in editing work.

**Introduction:**

1) Tobacco use is the leading cause of chronic obstructive pulmonary disease (COPD) (8,9) and the leading risk factor for respiratory health (10).

COMMENT: Unclear what is meant by this.

2) COPD affects up to 50% of older smokers, and smoking causes 80-90 percent of the morbidity associated with the illness (11,12).

COMMENT: Consider rephrasing this to express the risk group better.

3) Although no rapid or complete cure for COPD exists, early detection is crucial (13,14).

COMMENT: Rephrase to reflect the fact that there is no cure but the progression can slow down with certain health changes

4) Because of the smoking-induced irregular mucociliary system, the cilia's primary defense mechanisms will be impaired, and storing fluids and mucus may result in continuous cleaning of the airway surface (16).

COMMENT: Please clarify, the meaning of this is very unclear.

5) Many studies have indicated that smoking reduces lung volume and respiratory muscle strength, directly and negatively affecting respiratory system function (10,21-23), and is the leading cause of chronic obstructive pulmonary disease (7,22,24,25).

COMMENT: I don’t understand how smoking affects the respiratory muscle strength. This needs to be clarified so that the real meaning is expressed better.

**Methods**

Questions:

1. Why choose 2:1 ratio for the case-control groups sizes? Add explanation.
2. Were participants screened for environmental tobacoo smoke exposure? If so what was the criteria applied? I think this should be an important aspect to look at or at least discuss as a potential limitation of the study, if not considered.
6) The anthropometric measurements of height and weight were obtained using standardized measurement techniques using a stadiometer (KT-GFO6AKindcare- China) and a portable electronic bathroom scale (Omron HN-283-Japan).

COMMENT: I would like to read about the accuracy level here too rather than having to go and check the refs. Plse add the details here too.

**Data collection**

COMMENT: Overall in the methods I would like to see more detailed description of the methods. At the moments the methods are not written in the way that they could be followed to perform the work again. Changes and edits related to merely writing style but I do prefer more factual, detailed descriptions. Please consider rewriting the 2 paragraphs of the data collection at least.

7) The Fagerström Test for Nicotine Dependence (FTND) questionnaire

COMMENT: This is a tool and requires proper referencing. Pls edit.

8) Lung function tests were performed for all eligible subjects participating in the study with the PCbased Medikro® Pro (Finland) spirometer embedded with ambient condition measuring sensors for automatic real-time BTPS (body temperature, pressure, water vapour saturated) correction. The sensors of the spirometer tracked environmental conditions automatically and enabled real-time corrections. The results of the three best-breathing manoeuvres were recorded and printed for analysis. South Asian references were used.

COMMENT: How do you define 3 best breathing manouvers. Pls describe in details. ‘Best’ is unclear, and is different with different measurements. Please add details description.

9) Lung function measurements

COMMENT: Pls add a reference to the source doc where the methods of lung measurements are described.

**Results:**

Characteristics of participants

10) COMMENT: I would like to see the anthropometrics for male and females too. More informative. Pls consider adding the detail.

Table 1

11) COMMENT: Why was the Brinkman index used. I would like to see the reasoning in brief.

Table 3

12) COMMENT: Why not test for the difference using linear regression where you express the mean difference from the control (Non-smoker). This detail with confidence interval would be much more informative rather than just a cut-off of the Chi-square test.
13) Frequency of Cough. Intermittent/ continues

COMMENT: I would like to see a description of what these categories mean? And I would use persistent rather than continues as a category name.

Correlation of lung function parameters with smoking variables and anthropometric variables

14) A poor correlation was found between BMI and PEF values and

COMMENT: Pls finish off the sentence. Poor correlation is an unspecific term. Edit and clarify. Use non-significant instead if correlation is non-significant or weak to describe very low but significant correlation.

Table 5: Correlation of lung function parameters with smoking variables

15) COMMENT: Unsure why the anthropometrics are listed in the table. These are already in-built in the lung function measurements. Pls consider removing them. This applies to all tables where lung function parameters are expressed. Pls check all tables and edit accordingly.

**Discussion:**

16) Three hundred sixty daily male tobacco users and 180 non-smokers were randomly recruited for the study. The two groups had no significant difference in age or anthropometric features (p>0.05). The average daily cigarette usage of smokers was 5.73±4.88(SD), which is not a high frequency compared to many studies. The median values of smoking duration were 21.0±17.0(IQR) years, Brinkman index 80.0±135.0(IQR), and smoking-initiated age was 18.0 ±4.0 years. The age range of our participants was 21-60 years, and this age group was chosen by other studies as well (18,19). Cigarette usage in Asian nations is modest compared to the rest of the globe (18).

COMMENT: This is an interpretation and does not fit in the first paragraph of the discussion. Consider moving to an appropriate point in the latter part of the discussion.

17) COMMENT: I would like to see the following structure in the discussion.
I. First paragraph of the discussion should recap what this study aimed to do and summarise in brief the findings with minimal numeric detail.
II. Second paragraph should describe the weaknesses of the study and those results shown in the results section.
III. Third paragraph
IV. Interpretation and relating results to other findings and the research literature.
V. Limitations of the study
VI. Conclusions
